# Disease management programs for patients with type 2 diabetes mellitus in Germany: a longitudinal population-based descriptive study

**DOI:** 10.1186/s13098-017-0236-y

**Published:** 2017-05-18

**Authors:** Michael Mehring, Ewan Donnachie, Florian Cornelius Bonke, Christoph Werner, Antonius Schneider

**Affiliations:** 1 Institute of General Practice, Technische Universität München, Orleansstraße 47, 81667 Munich, Germany; 2 Association of Statutory Health Insurance Physicians of Bavaria, Elsenheimerstraße 39, 80687 Munich, Germany

**Keywords:** Disease management program, Type 2 diabetes, Active patient participation, Self-management, Guideline care, General practice

## Abstract

**Background:**

The primary aim of the disease management program (DMP) for patients with diabetes mellitus type 2 is to improve the quality of health care and the treatment process. 12 years after its introduction in Germany, there is still no consensus as to whether DMP has been effective in reaching these goals.

**Methods:**

A retrospective longitudinal population-based study between 2004 and 2015 were conducted to evaluate the DMP for type 2 diabetes in Bavaria using routinely collected patient medical records hold from the National Association of Statutory Health Insurance Physicians of Bavaria.

**Results:**

During the first 12 years of DMP, the number of participants increased continually to reach 580,222 in 2015. The proportion of participants older than 70 years increased during the observation from 41.6 to 51.1%. The percentage of smokers increased slightly from 9 to 11%. Similarly, the distribution of body mass index remained constant with approximately 50% of patients having a body mass index >30 kg/m^2^. Control of HbA1c was without an appreciable change over the course, with between 8.3 and 9.4% of all patients with uncontrolled values higher than 8.5%. Prescription of metformin increased from 40.5% in 2004 to 54.1% in 2015. Among patients receiving insulin, the proportion receiving a combined therapy with metformin increased from 28.4% in 2004 to 50.8% in 2015. In contrast, the percentage with insulin monotherapy decreased from 55.4 to 33.7%. The proportion of patients with a diabetic education increased within the course from 12.8 to 29.3%.

**Conclusion:**

Data from the German DMP for type 2 diabetes demonstrates an improvement in the quality of care with respect to pharmacotherapy and patient education and therefore to an improved adherence to guidelines. However, no appreciable improvement was observed with regard to smoking status, obesity or HbA1c control.

## Background

Chronic diseases are one of the main causes of increased morbidity and mortality risk worldwide [1]. Diabetes mellitus was once a disease of concern almost exclusively in developed western industrial nations, but is now also increasingly an issue in developing countries. Worldwide, the number of adults with diabetes worldwide has more than doubled in the last 3 decades [2]. Diabetes mellitus is a chronic disease often associated with serious complications such as retinopathy, nephropathy, neuropathy, ischemic heart disease, peripheral vascular disease and cerebrovascular disease. Its global burden to public health systems and high potential for a deep impact on economies worldwide motivate further research to improve the management of patients with diabetes mellitus.

In Germany, disease management programs (DMP) for diabetes and other chronic conditions were introduced between 2003 and 2007. The aim was to improve the quality of health care and the treatment process [3]. Currently, more than 7.7 million statutorily insured patients in Germany are enrolled in one of the six disease management programs [4]. As of present, there are DMPs for patients with breast cancer, diabetes type 1 and type 2, coronary heart disease (CHD), asthma and chronic obstructive pulmonary disease (COPD). Further DMPs are currently being planned for patients with chronic heart failure, depression, and chronic back pain. Although 4.04 million statutorily insured patients took part in one of 1.723 registered DMPs for type 2 diabetes in 2015, it is still highly debated how effective these programs in Germany are [5] and if they have achieved their goals. The main reason for this nescience is that the DMP were introduced at a national level without incorporating a valid randomized or pseudo-experimental evaluation design [6]. Besides the methodological issues, interpretation of the available findings is further complicated by conflicting interests, for example due to the initial coupling of the DMP with the financial risk adjustment scheme for health care insurances. For this reason, we limit ourselves to a purely descriptive analysis of the DMP between 2004 until 2015 in order to assess how the structure and treatment of this patient collective has developed.

A central intention of the German DMP was to introduce a data-driven system for continuous quality improvement [7]. For evaluation and quality improvement purposes relevant data on each patient is collected in a standardized procedure. The present investigation therefore assesses whether key indicators for quality improved during the first 12 years of DMP in Bavaria. In general studies investigating the utility of type 2 diabetes mellitus DMPs have come to varying conclusions. Some of these studies suggest that the German DMPs have improved the quality of care [8, 9]. Other studies showed no improvements for DMP-diabetes participants [10, 11].

## Methods

### The German DMP for diabetes mellitus type 2

In 2001 a committee of experts reporting to the German Federal Minister of Health criticized what they had identified as deficits in routine care of chronically ill patients, including those with diabetes mellitus type 2 [12]. A DMP was suggested as a quality program to facilitate the continuous improvement of this care. In the end the DMP for diabetes mellitus type 2 was accredited by the German Federal Insurance Agency (German: *Bundesversicherungsamt*) in 2002 and introduced in Bavaria in July 2004. Its aim is to improve long-term care by establishing standards for diagnosis, treatment, documentation, quality assurance and referral, whilst requiring active patient participation. In parallel to the introduction of DMP, the national diabetes mellitus type 2 guideline [13] was developed and brought into effect as a guideline for the German health care system. In order to enroll a patient into the DMP diabetes mellitus type 2, the diagnosis needs to be confirmed and documented by the coordinating general practitioner according to established criteria. Participating patients receive a quarterly or half-yearly check-up by their coordinating GP, with the interval decided by the physician based on symptom severity and overall patient health. A centralized reminder system for patients and practices helps to ensure that these regular consultations are not overlooked. Health insurance companies support their patients with structured information to assist self-management and by providing other insurer-specific incentives (e.g. until its abolition at the end of 2012, a quarterly consultation fee of €10 was waived for DMP patients). Physicians commit to treat patients according to evidence-based guidelines. To this end, a standardized medical record is completed at each check and submitted to various official agencies for quality assurance purposes. This file contains details of the physical examination (vital parameters and comprehensive foot examination including pulses), HbA1c, presence of albuminuria, medical history, diabetes related and antihypertensive medication, patient education for diabetes and hypertension, a patient-specific HbA1c target agreement, documentation of hospitalization or emergency treatment and referrals to a diabetologist or other specialist. The DMP diabetes mellitus type 2 was underpinned by the introduction of additional quality improvement measures. GPs receive half-yearly feedback reports to benchmark their performance on the basis of agreed quality indicators (e.g. percentage of patients with an HbA1c >8.5%). Additionally, participating GPs are obliged to complete continuous diabetes-specific medical education at least once every 3 years. These medical education programs are provided by various commercial and non-profit organizations including the National Association of Statutory Health Insurance Physicians of Bavaria (German: *Kassenärztliche Vereinigung Bayerns—KVB*). Finally, the KVB utilizes CME events and its members’ journal to engage coordinating physicians in the process of quality improvement.

### Statistical evaluation

Patients medical records in pseudonymised form were analyzed by the National Association of Statutory Health Insurance Physicians of Bavaria. A retrospective longitudinal population-based study was conducted between 2004 and 2015. The data were analysed in a pure descriptive manner. Statistical analysis was conducted using the R environment for statistical computing [14].

## Results

Since the introduction of DMP, the number of participating patients has increased steadily. Whereas in the first year 2004, a total of 249,227 patients were enrolled, this number has more than doubled over the first 12 years (Table 1). While the distribution of gender remained constant over the entire observation period, the age distribution of the DMP collective increased steadily. The percentage of smokers increased slightly up to 11.2% in 2015. Similarly, no appreciable change can be observed in BMI. The percentage of patients with a BMI between 18.5 and 29.9 kg/m^2^ decreased slightly, while the percentage with BMI ≥35 kg/m^2^ increased and the group between 30 and 34.9 kg/m^2^ showed no substantial change. The numbers of participating physicians increased from 5525 at program begin in 2004 to 8257 in 2015. These are predominantly general practitioners (97%), followed by internists, diabetologists or endocrinologists (1.2%) and other physicians in private practices (0.7%).

**Table 1:** Patients baseline data

	2004	2005	2006	2007	2008	2009	2010	2011	2012	2013	2014	2015
No. patients												
n	272,230	322,407	353,818	405,495	443,943	475,418	506,221	532,394	557,679	565,262	574,249	580,222
Age												
0–30												
n	603	619	684	893	1072	1189	1298	1473	1589	1594	1665	1678
%	0.2	0.2	0.2	0.2	0.2	0.3	0.3	0.3	0.3	0.3	0.3	0.3
31–40												
n	3488	4086	4519	5140	5698	6165	6496	6828	7195	7221	7602	7846
%	1.3	1.3	1.3	1.3	1.3	1.3	1.3	1.3	1.3	1.3	1.3	1.4
41–50												
n	15,658	18,902	20,723	23,847	26,157	28,523	30,524	32,418	33,870	33,973	33,838	33,875
%	5.8	5.9	5.9	5.9	5.9	6.0	6.0	6.1	6.1	6.0	5.9	5.8
51–60												
n	44,330	53,046	59,448	68,438	73,860	77,242	80,621	83,833	86,929	87,328	89,379	91,179
%	16.3	16.5	16.8	16.9	16.6	16.2	15.9	15.7	15.6	15.4	15.6	15.7
61–70												
n	94,715	108,701	114,317	126,291	134,234	138,947	142,553	143,662	146,125	147,232	148,731	149,458
%	34.8	33.7	32.3	31.1	30.2	29.2	28.2	27.0	26.2	26.0	25.9	25.8
71–80												
n	81,740	97,102	108,561	126,700	141,195	154,850	169,104	181,978	192,709	194,937	196,654	195,301
%	30.0	30.1	30.7	31.2	31.8	32.6	33.4	34.2	34.6	34.5	34.2	33.7
80+												
n	31,696	39,951	45,566	54,186	61,727	68,502	75,625	82,202	89,262	92,977	96,380	100,885
%	11.6	12.4	12.9	13.4	13.9	14.4	14.9	15.4	16.0	16.4	16.8	17.4
Female gender												
n	106,443	139,086	162,025	193,781	220,973	241,668	257,076	270,660	283,446	286,676	290,607	292,302
%	51.5	51.3	51.2	51.1	50.9	50.9	50.8	50.9	50.8	50.7	50.6	50.4
Smoker												
n	24,544	27,147	29,236	33,277	39,754	44,172	48,549	53,115	57,421	59,631	62,545	64,959
%	9.0	8.4	8.3	8.2	9.0	9.3	9.6	10.0	10.3	10.5	10.9	11.2
BMI												
0–18.5												
n	446	176	149	147	1532	1794	1845	1935	2029	2080	2208	2178
%	0.6	0.4	0.4	0.4	0.4	0.4	0.4	0.4	0.4	0.4	0.4	0.4
18.5–24.9												
n	9782	5202	4848	4794	52,452	58,783	61,942	64,878	67,348	68,076	68,603	69,645
%	13.7	12.5	12.3	12.7	12.5	12.5	12.3	12.2	12.1	12.1	12.0	12.0
25.0–29.9												
n	28,650	16,202	15,056	14,162	160,124	178,845	189,735	198,157	206,727	208,359	210,000	210,317
%	40.0	39.1	38.2	37.5	38.3	38.0	37.7	37.4	37.2	37.0	36.7	36.3
30.0–34.9												
n	20,872	12,312	11,726	11,008	125,014	140,792	150,947	159,081	166,511	168,794	171,675	173,238
%	29.1	29.7	29.7	29.2	29.9	29.9	30.0	30.0	30.0	29.9	30.0	29.9
35.0–39.9												
n	8101	5033	4991	4903	52,728	60,226	65,537	70,023	74,078	75,821	77,638	79,152
%	11.3	12.1	12.7	13.0	12.6	12.8	13.0	13.2	13.3	13.5	13.6	13.7
40+												
n	3805	2557	2677	2728	26,168	30,646	33,898	36,464	39,052	40,550	42,669	44,260
HbA1c >8.5%												
n	22,821	28,588	30,563	36,343	36,264	42,762	47,335	49,624	52,187	50,426	47,825	47,951
%	8.4	8.9	8.6	9.0	8.2	9.0	9.4	9.3	9.4	8.9	8.3	8.3
Patient education												
n	34,840	73,454	89,348	106,870	125,175	137,569	147,672	156,205	162,850	165,923	168,197	169,877
%	12.8	22.8	25.3	26.4	28.2	28.9	29.2	29.3	29.2	29.4	29.3	29.3

DMP diabetes mellitus Typ 2: 2004–2015

**Fig. 1:**
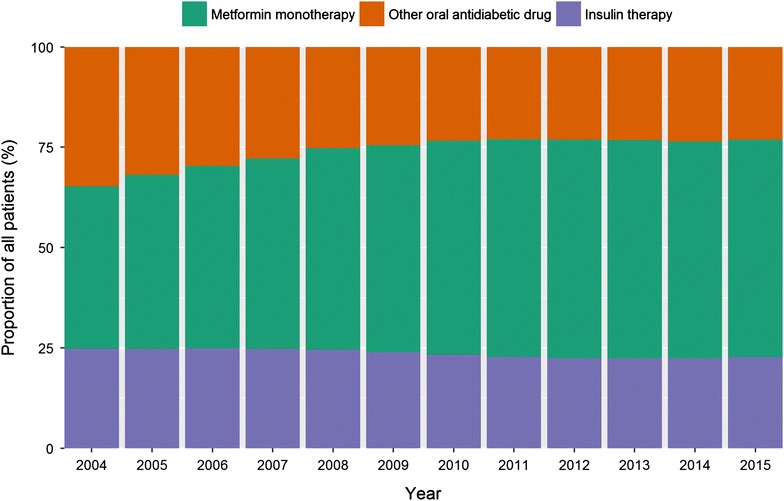
Distribution of prescribed medication by 2004–2015

The analysis of the prescribed medication reveals a number of distinct findings (Table 2). The most imposing result is a clear increasing trend in the prescription of metformin. Whereas in 2004, 40.5% of all patients were prescribed metformin, this share increased to 54.1% in 2015 (Fig. 1).

**Table 2:** Medication

	2004	2005	2006	2007	2008	2009	2010	2011	2012	2013	2014	2015
Antihyperglycemic medication												
n	206,015	246,453	270,251	309,867	344,343	367,046	390,126	406,919	421,065	422,019	423,640	425,342
%	75.7	76.4	76.4	76.4	77.6	77.2	77.1	76.4	75.5	74.7	73.8	73.3
Metformin												
n	110,237	139,903	160,445	192,346	223,018	245,607	269,810	288,871	303,841	307,339	310,177	314,075
%	40.5	43.4	45.3	47.4	50.2	51.7	53.3	54.3	54.5	54.4	54.0	54.1
Insulin												
n	67,491	79,835	88,270	100,335	108,872	113,476	117,963	121,185	125,045	126,496	128,680	131,760
%	24.8	24.8	24.9	24.7	24.5	23.9	23.3	22.8	22.4	22.4	22.4	22.7
Of which												
Monotherapy												
n	37,378	42,517	45,574	50,090	51,680	51,583	51,011	50,229	49,289	47,134	45,397	44,347
%	55.4	53.3	51.6	49.9	47.5	45.5	43.2	41.4	39.4	37.3	35.3	33.7
With metformin												
n	19,182	24,691	29,586	35,946	41,452	45,734	50,160	53,992	58,085	60,871	63,473	66,900
%	28.4	30.9	33.5	35.8	38.1	40.3	42.5	44.6	46.5	48.1	49.3	50.8

DMP diabetes mellitus Typ 2: 2004–2015

Simultaneously, the prescription of insulin decreased slightly over the course from 24.8% in 2004 to 22.7% in 2015. Additionally the use of an insulin monotherapy declined significantly from 55.4% in 2004 to 33.7% in 2015, the combination of metformin and insulin increased steadily from 28.4 to 50.8%. The proportion of patients with a successfully completed diabetic education increased within this period from 12.8 to 29.3%. The proportion of patients with an HbA1c value higher than 8.5% showed over the course a marginal short-term increase from 8.4 to 9.4%, decreasing again to 8.3% of all patients in 2015.

**Fig. 2:**
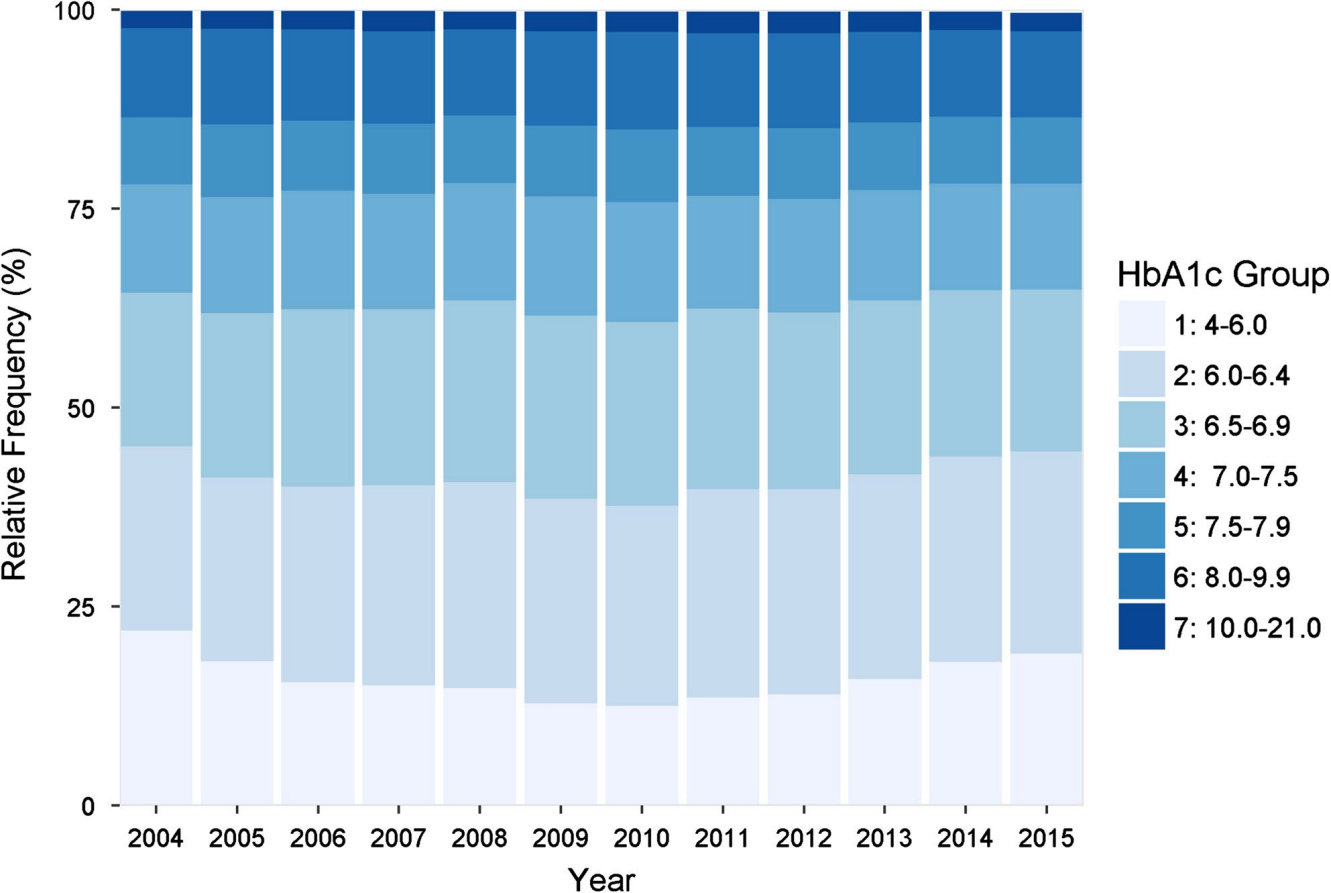
Distribution of grouped HbA1c values by 2004–2015

Figure 2 shows the distribution of grouped HbA1c values by year. Over the entire course of the observation, the proportion of patients with an HbA1c value above 8.0% remained approximately constant at around 20%. The proportion of patients with HbA1c below 6% exhibits a U-shaped development, decreasing from 22 to 12% by 2010 and then increasing to 20% of patients by 2015.

## Discussion

The main results of the present evaluation are increased prescription rates of metformin and the combination of metformin and insulin since the implementation of DMP for diabetes in Bavaria in 2004. At the same time, the proportion receiving insulin decreased and, among these patients, insulin monotherapy became less common.

Since the early 1990s, disease management programs for diabetes mellitus have been implemented in many countries to improve quality of life and treatment process and to reduce healthcare expenditures. In Germany, the DMP for patients with diabetes mellitus type 2 was introduced nationwide starting in 2002. However, an adequate evaluation scheme, for example by means of a cluster-randomized controlled trial, was not implemented and so a retrospective causal analysis is difficult to perform and necessarily limited. A comprehensive program evaluation requires a control group design. We therefore present a purely descriptive analysis charting the development of the program between 2004 and 2015. Previous findings revealed already for example within an observational study of 11,079 patients over 3 years an association between reduced mortality and the participation in a German DMP for diabetes mellitus type 2 [9]. Laxy et al. [15] found a clear positive impact of guideline care and increased self-management for patients within a DMP. A recent published evaluation of the Austrian DMP for patients with type 2 diabetes mellitus showed within a propensity score matching analysis a clear survival and cost benefit for DMP participants compared to non-participants [16]. Another recent findings showed that the participation of a German DMP has a positive impact on HbA1c values [17]. Sönnichsen et al. found within a cluster randomized trial that the process quality enhances of DMP participants without an improvement of the metabolic control [18]. Additionally a recent systemic literature review [19] from German DMPs for type 2 diabetes found besides a lower overall mortality also an improvement in process parameters from DMP participants. Some of the already existing results are in line and were largely corroborated by our present descriptive findings. The present findings are solely descriptive and do not raise the claim to prove the above mentioned associations, but some of the previous methodological limited findings from the German DMP were supported by our present descriptive results.

In particular the increasing prescription of metformin reflects a stronger adherence to guidelines, with metformin almost universally recommended as a first-line drug treatment [13, 20]. This is justified by good tolerance, few side effects, a decrease in HbA1_**C**_ by 1.5–2%, avoidance of hypoglycaemia, decrease in body mass index, proven positive effect on cardiovascular complications and mortality, high therapy compliance rate and low treatment costs. Additionally the improvement of the diabetes education reflects an initial increase in guideline adherence, but the almost unchanged saturation level between 2011 and 2015 suggests that further efforts are needed to promote patient education. The observed improvements in diabetes care may conceivably have been achieved by the accompanying quality improvement strategies as outlined in the methods section. Individual feedback reports and medical education schemes are known to be effective to improve the quality of chronic care [21, 22]. However, a further development and support of establishing standards for diagnosis, treatment, documentation, quality assurance, and enhancing active patient participation is still desirable and in the sense of a better patient care.

Two previous reviews [23, 24] concluded that a DMP lead to a modest extent to an improvement of a glycemic control. Otherwise, a systematic literature review, conducted in 2012 came to the conclusion that the analyses regarding the effectiveness of DMPs were not feasible due to heterogeneity of study designs [25]. The present results in regard to the glycemic control are hard to interpret without a comparison group. The mainly unchanged HbA1c values over the course exclude at least a serious aggravation of the metabolic control. However, it is unclear whether our findings indicate an improvement in glycemic control.

The main limitation of the present evaluation and indeed of almost all studies relating to the German DMP are its purely descriptive nature and the absence of a suitable control group and so the missing comparability of DMP diabetes and standard care regarding their effectiveness. This might lead to a selection bias towards more motivated and healthier patients participating in a DMP. Additionally, systematic differences may exist between those GPs participating in the program and those who, for a variety of reasons, do not take part. On the other hand, the participation of over 580,000 patients provides an almost unrivalled data source with which to evaluate the quality of care within DMP. This enables us to conclude with some certainty that the first 12 years of DMP in Bavaria have seen ongoing improvement in pharmacotherapy and guidelines adherence, hence also an overall improvement in treatment process for patients with diabetes mellitus type 2.

## Conclusion

Summarizing all results leads to the suggestion that the German DMP for type 2 diabetes has been effective in enhancing the quality of care in regard to an improved pharmacotherapy and patient education and therefore to an improved adherence to guidelines. However, no appreciable improvement was observed with regard to smoking status, obesity or HbA1c control.
